# A 19 Year Analysis of Small Mammals Associated with Human Hantavirus Cases in Chile

**DOI:** 10.3390/v11090848

**Published:** 2019-09-12

**Authors:** Fernando Torres-Pérez, R. Eduardo Palma, Dusan Boric-Bargetto, Cecilia Vial, Marcela Ferrés, Pablo A. Vial, Constanza Martínez-Valdebenito, Carlos Pavletic, Alonso Parra, Pablo A. Marquet, Gregory J. Mertz

**Affiliations:** 1 Instituto de Biología, Pontificia Universidad Católica de Valparaíso, Valparaíso 2373223, Chile; dboricbargetto@gmail.com; 2 Laboratorio de Biología Evolutiva, Departamento de Ecología, Pontificia Universidad Católica de Chile; Santiago 8331150, Chile; epalma@bio.puc.cl; 3 Programa Hantavirus, Instituto de Ciencias e Innovación en Medicina, Facultad de Medicina, Clínica Alemana, Universidad del Desarrollo, Santiago 7610658, Chile; mcvial@udd.cl; 4 Laboratorio de Infectología y Virología Molecular, Red Salud UC-Christus, Departamento de Enfermedades Infecciosas e Inmunología Pediátricas, Pontificia Universidad Católica de Chile, Santiago 8330024, Chile; marceferres@gmail.com (M.F.); constanza.martinez.v@gmail.com (C.M.-V.); 5 Instituto de Ciencias e Innovación en Medicina, Facultad de Medicina, Clínica Alemana, Universidad del Desarrollo, Santiago 7610658, Chile; pvial@udd.cl; 6 Oficina de Zoonosis y Control de Vectores, División de Políticas Publicas Saludables y Promoción, Subsecretaría de Salud Pública, Ministerio de Salud, Santiago 8320064, Chile; alonsoparra@minsal.cl; 7 Departamento de Ecología, Pontificia Universidad Católica de Chile, Santiago 8331150, Chile; pmarquet@bio.puc.cl; 8 Division of Infectious Diseases, Department of Internal Medicine, University of New Mexico, Albuquerque 87131, New Mexico; gmertz@salud.unm.edu

**Keywords:** *Andes orthohantavirus*, Chile, hantavirus cardiopulmonary syndrome, hantavirus spatial distribution, *Oligoryzomys longicaudatus*, peridomestic, rodent reservoir, seroprevalence

## Abstract

Small mammals present in areas where hantavirus cardiopulmonary syndrome (HCPS) cases had occurred in central and southern Chile were captured and analyzed to evaluate the abundance of rodents and seroprevalence rates of antibodies to *Andes orthohantavirus* (ANDV). Sampling areas ranged from the Coquimbo to Aysén regions (30–45° S approx.) regions. Ninety-two sites in peridomestic and countryside areas were evaluated in 19 years of sampling. An antibody against ANDV was detected by strip immunoassay in 58 of 1847 specimens captured using Sherman traps. Of the eleven species of rodents sampled, *Abrothrix olivacea, Oligoryzomys longicaudatus* and *Abrothrix hirta* were the most frequently trapped. *O. longicaudatus* had the highest seropositivity rate, and by logistic regression analysis, *O. longicaudatus* of at least 60 g had 80% or higher probability to be seropositive. Sex, age and wounds were significantly related to seropositivity only for *O. longicaudatus.* Across administrative regions, the highest seropositivity was found in the El Maule region (34.8–36.2° S), and the highest number of HCPS cases was registered in the Aysén region. Our results highlight the importance of long term and geographically extended studies, particularly for highly fluctuating pathogens and their reservoirs, to understand the implications of the dynamics and transmission of zoonotic diseases in human populations.

## 1. Introduction

Emerging zoonotic diseases are a major concern for public health systems, producing new or previously unrecognized diseases, or rapidly increasing incidence of infection in the human population. Ecological, environmental changes or changes in human demographics and behavior, which increase chances of contact with reservoirs, are the main factors responsible for these emergences [1]. RNA viruses represent an important group of emerging human pathogens, including several harbored by animal reservoirs [2]. Recent examples are the outbreak of pandemic influenza A subtype H1N1, human immunodeficiency virus, Ebola virus, hepatitis C virus, West Nile virus, rabies virus and severe adult respiratory distress (SARS), which have challenged control and prevention measures of health systems.

Orthohantaviruses are segmented negative-strand RNA viruses of the family *Hantaviridae*, which contain large (L), medium (M) and small (S) segments encoding RNA-dependent RNA polymerase, glycoproteins Gn and Gc and nucleocapsid (N) protein, respectively [3]. Several orthohantavirus strains are recognized as human pathogens [4,5,6]. Orthohantavirus infections became a concern in the Americas in 1993 after detecting an outbreak of a previously unrecognized syndrome [7]. The disease (hantavirus cardiopulmonary syndrome, HCPS), was produced by infection with the newly-discovered Sin Nombre virus (SNV), with *Peromyscus maniculatus* (deer mouse) identified as the reservoir [8]. Since then, more than forty-three genotypes have been reported in the Americas alone [5], with nearly half of those associated with clinical cases of HCPS. Pathogenic hantaviruses are carried by rodents of the families Muridae and Cricetidae, while hantaviruses of unknown pathogenicity have also been discovered in shrews, moles and bats [9,10,11,12,13]. Hantavirus infection is transmitted to humans via inhalation of contaminated rodent excreta and secretions or less commonly through rodent bites [5]; the case fatality rate in the Americas is around 36% [6,14,15,16], although it may reach up to 56% [17].

In southern South America, *Andes orthohantavirus* (ANDV) is a major etiologic agent of HCPS, and the sigmodontine rodent *Oligoryzomys longicaudatus* (the pigmy rice rat) is the reservoir [18,19]. *O. longicaudatus* is distributed in Chile and Argentina, with higher abundances in mesic areas in the temperate forests and Patagonia [20]. Spillover has been reported to other sympatric rodent species, although with no apparent epidemiological importance [21]. Strikingly, person-to-person transmission of hantavirus infection has only been demonstrated for ANDV [16,22,23,24,25].

In Chile, 1157 cases have been confirmed between 1995 and December 2018 in 11 administrative regions from the Coquimbo region in the north to the Aysén region in the south (30–45° S approx.; epi.minsal.cl). Although human infections have been reported in a variety of environments, higher numbers of HCPS cases tend to be associated with people living, working or visiting rural areas where the reservoir occurs [20,26]. The settlement of human populations in areas near habitats where wild infected rodents occur increases the likelihood of contact and the probability of contracting the infection. Although the relation between the number of infected hosts and human incidence depends on complex ecological processes [27], there is evidence suggesting that higher seroprevalence in the hantavirus reservoir may lead to more human infections [28]. To understand eco-epidemiological processes that might be responsible for human hantavirus infections, we undertook an analysis of the abundance and seroprevalence of small mammals in Chile in peridomestic and countryside locations where cases of HCPS had occurred. To date, *O. longicaudatus* is the sole recognized reservoir for ANDV in Chile, and only a few studies have explored the participation of other small mammal species in the transmission of the disease [19,29]. Our study highlights the value of combining information from reservoir ecology, epidemiology and geography to gain insights into the persistence and transmission of zoonotic diseases.

## 2. Materials and Methods

### 2.1. Ethical Statement

Permission to trap small mammals was obtained from the Servicio Agrícola y Ganadero (SAG, Chile; permits 4560/2018, 5346/2016, 3432/2013, 7445/2013, 1607-2012, 6134-2011, 1158/2011, 17/2000, 7325/2005, 1056/1999), and Corporación Nacional Forestal (CONAF, Chile; permits 10-02/2002, 13-03/2003, 14-99/2004, 24/2004, 07-06/2006). All the National Institute of Health studies were approved by the Institutional Animal Care and Use Committee (IACUC) of the University of New Mexico Health Sciences Center under protocol number 14-101118-Field-HSC, and the Department of Health and Human Services of the National Institute of Health, Animal Welfare Assurance A5848-01. Small mammals trapping protocols and biosafety procedures for CONICYT-FONDECYT and CONICYTY-PIA Anillo studies (Chile), were reviewed and approved by institutional ethics and biosafety review boards at the Pontificia Universidad Católica de Valparaíso (certifications CBPUCV 22.04.2011, CBPUCV 11.2015, CBPUCV 06.2017), and the Pontificia Universidad Católica de Chile (CBB 7/8/2005; CBB 181/2010; CBB-143/2011).

### 2.2. Sampling

Sampling was conducted in those areas where human cases of HCPS were confirmed through the Chilean Ministry of Health (see below). When more than one case was reported simultaneously, we chose that site that was not sampled previously and/or involved more people that contracted the disease. Sampling was performed within eight weeks after the onset of symptoms in the index case. We also chose sampling sites according to the following characteristics: Availability of sampling execution, sites where vegetation characteristics would be part of the habitat of the primary reservoir rodent *O. longicaudatus*, and sites where the local population reported seeing rodents or rodent droppings. The study covered rural, urban and periurban areas [20] ranging from Chiñigue, Coquimbo region (30°30’46.02”S, 71°6’8.46”W) to Cerro Negro, Aysén region (45°35’0.48”S, 71°56’25.5”W), 1675 km north to south. To capture small mammals, standard live Sherman traps (8 cm × 9 cm × 23 cm) were used. Traps were located in forests, meadows, plantations and along fences and roads [20,30]. Traps were installed for three consecutive nights varying between 120–771 traps/night. For the capture and manipulation of small mammals, the processing and handling standards were followed according to the protocols of the Center for Infectious Diseases and Prevention of Atlanta [30,31] and the American Society of Mammalogists [32]. Species identification followed specialized literature. We also followed the recent description of *A. hirta* (35°S to north of Tierra del Fuego) as sister species of *A. longipilis* (distributed between 30°S to 35°S approx.) [33,34].

### 2.3. Relative Abundance

Relative abundance was determined for all captured species (*O. longicaudatus, A. olivacea*, *A. longipilis*, *A. hirta*, *A. sanborni*, *A. manni*, *L. micropus*, *P. darwini*, *M. musculus*, *R. norvegicus* and *R. rattus)* between years 2000 and 2018. Each sampling site was categorized by locality (including county), administrative region (from the Coquimbo to Aysén), sampling year and coordinates. The relative abundance for each species and sampling site was measured by the number of individuals captured per species/total number of traps) × 100 [20,30].

### 2.4. Relative Seropositivity

Small mammals captured were anesthetized and a blood sample was taken from the retro-orbital sinus by means of a heparinized capillary, or by cardiac puncture. Blood samples were placed in cryovials and preserved in liquid nitrogen and brought to the laboratory for analysis. In addition, for each specimen the heart, kidney, spleen, liver and lung were extracted, and stored in liquid nitrogen. Voucher specimens were preserved in 96% ethanol, and deposited in the Colección de Flora y Fauna, Pontificia Universidad Católica de Valparaíso (Valparaíso, Chile), Colección de Flora y Fauna, Professor Patricio Sánchez Reyes, Pontificia Universidad Católica de Chile (Santiago, Chile) and in the Division of Mammals of the Museum of Southwestern Biology, University of New Mexico (Albuquerque, NM). For the determination of antibodies against ANDV, the SIA (strip immunoassay vacuum-blot test) technique was used following the method described previously (2004) [20,35].

To determine the relative intraspecific seropositivity for hantavirus by locality, the presence of antibodies to the virus was standardized using the ratio between the number of seropositive at each sampled locality and the trapping success (number of captures by number of total traps-nights). All values were determined for *O. longicaudatus*, *A. hirta* and *A. olivacea* that resulted positive for antibodies against the ANDV [36]. In addition, the total relative seropositivity by sampling site (total seropositives of the locality/trapping effort) and by region (total seropositives of the region/trapping effort) was estimated.

### 2.5. Confirmed Human Cases of Hantavirus

The confirmed human cases of hantavirus between 2000 and 2018 were obtained using two sources of information: (i) Through a national research network established by the Hantavirus Program in which HCPS cases were enrolled in clinical research studies shortly after hospital admission, and (ii) through the National Notification report of the Ministry of Health. HCPS is a disease that must be reported as soon as the diagnosis is suspected. Human cases were classified by locality and the administrative region.

### 2.6. Ecological Features of the Reservoirs

To determine the relationship between seropositives and age, small mammals were sexed and classified by age into juveniles or adults according to their body mass and reproductive status (i.e., males with scrotal or abdominal testes and females with or without perforated vagina, pregnant and/or lactogenic) [37,38]. We also registered presence or absence of wounds or scars, such as bites on the ears or tail, to determine the relationship between seropositives and wounds.

### 2.7. Data Analysis and Statistics

The χ2 test and Fisher’s exact test were used to compare the following categorical variables between groups using 2 × 2 tables: Serostatus (seropositive or seronegative) according to sex (male or female), age (adult or juvenile) and wounds (injured or not injured). The significance criterion was set at *p* < 0.05. Calculations were performed using the VassarStats website for statistical computation (http://www.vassarstats.net). The effect of the weight (independent variable) on the serostatus (response variable) was analyzed by using a logistic regression performed within the free data analysis environment R [39].

### 2.8. Serology and Human Hantavirus Cases

We determined the rodents’ relative seropositivity in sites associated with human hantavirus cases, and human hantavirus cases across the 11 sampled administrative regions in Chile. The numbers of human hantavirus cases were standardized to cases per 1000 rural inhabitants (information extracted from the National Institute of Statistic of Chile, www.ine.cl).

## 3. Results

### 3.1. Rodents Relative Abundance

**Figure 1:**
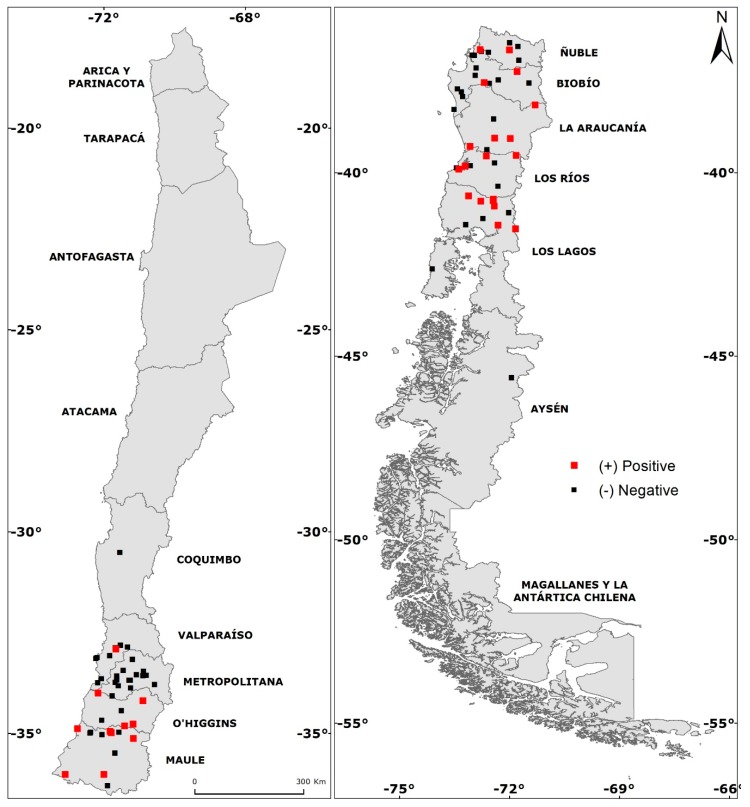
Small mammal trapping sites associated with human hantavirus cases sampled in the nineteen-year study across administrative regions in Chile. Sites with small mammals seropositive for anti-*Andes orthohantavirus* (ANDV) antibodies are shown in red. Sites without seropositive rodents are shown in black.

During nineteen years of sampling (2000–2018), ninety-two sites associated with human hantavirus cases between Coquimbo and Aysén (30–45° S approx.) regions in Chile were studied for the presence of rodents (Figure 1). We trapped a total of 1847 specimens of eleven species of rodents. The sigmodontine species captured were *Oligoryzomys longicaudatus* (*N* = 631), *Abrothrix olivacea* (*N* = 636), *Abrothrix longipilis* (*N* = 43), *Abrothrix hirta* (*N* = 234), *Abrothrix sanborni* (*N* = 9)*, Abrothrix manni* (*N* = 8), *Loxodontomys micropus* (*N* = 18) and *Phyllotis darwini* (*N* = 18). Murine rodents captured were *Mus musculus* (*N* = 52)*, Rattus norvegicus* (*N* = 95) and *Rattus rattus* (*N* = 103). Overall, rodents were captured in all sampling sites, and seropositive specimens were found in 30 of the 92 sites (see below).

Relative abundance (Table 1, Appendix A) of rodents ranged between 17.5 (*O. longicaudatus,* Ñancul, Panguipulli, Los Ríos region) to 0.13 (*A. hirta*, *L. micropus* and *R. rattus*; Las Quemas, Puerto Montt, Los Lagos region). The highest relative abundance for *O. longicaudatus* was found in Ñancul, Panguipulli, Los Ríos region (17.5), and the lowest was found in Las Peñas 2002, San Fernando, O’Higgins region (0.28). For *A. olivacea*, the highest relative abundance was found in Alto Las Viñas, Los Ángeles, Biobío region (8.67), and the lowest value was 0.15 in Tres Esquinas, Coihueco (Ñuble region). Relative abundance for *A. longipilis* ranged from 4.0 in Fundo La Ventolera, Santo Domingo (Valparaíso region) to 0.33 in Quilamuta-Las Cabras (O’Higgins region). For *A. hirta*, the highest value of relative abundance was in Vilumanque, Concepción (Biobío region; 8.72), and the lowest relative abundance was in Las Quemas, Puerto Montt (Los Lagos region; 0.13). For *A. sanborni* the highest value of relative abundance was in Chiloé National Park (Los Lagos region; 1.04), and the lowest relative abundance was in Fundo Futangue, Lago Ranco (Los Ríos region; 0.73). Relative abundance for *L. micropus* ranged from 1.67 in Llanquén, Lonquimay (Araucanía region) to 0.13 in Las Quemas, Puerto Montt (Los Lagos region). For *P. darwini*, values ranged from 1.38 in Coya, Machalí (O’Higgins region) to 0.33 in Las Peñas 2018 (O’Higgins region). Among murine rodents, values ranged between 2.53 (Llaillay, Valparaíso region) to 0.15 (Los Maitenes, Lolol, O’Higgins region) in *Mus musculus*. Similar values of relative seropositivity were found for both *Rattus* species: For *R. norvegicus* values ranged from 3.45 in Coya (O’Higgins region) to 0.15 in Chiloé National Park (Los Lagos region), and for *R. rattus* values ranged from 3.55 in Boroa Norte, Toltén (Araucanía region) to 0.13 in Las Quemas, Puerto Montt (Los Lagos region).

### 3.2. Total Seropositivity

**Figure 2:**
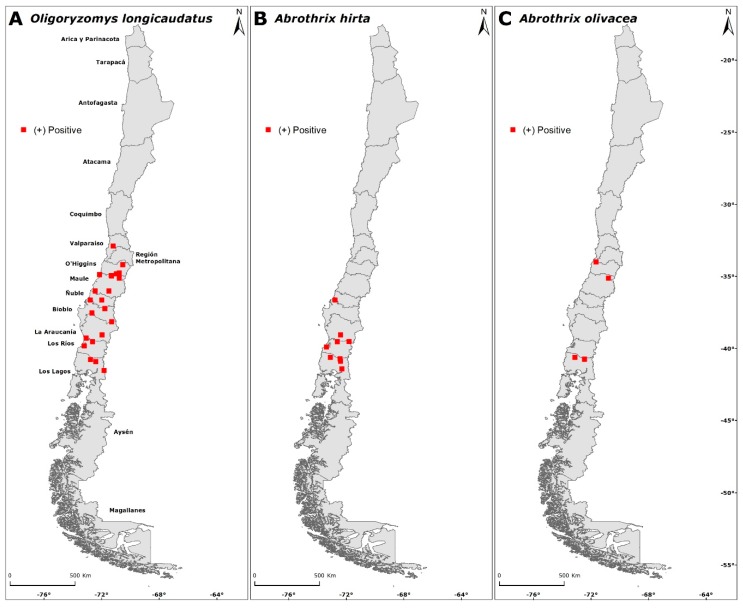
Distribution of rodents with anti-ANDV antibodies in areas associated with human hantavirus cases for *O. longicaudatus*, *A. hirta* and *A. olivacea* during the nineteen-year study in Chile.

Globally, of the 1847 small mammals analyzed for antibodies against the ANDV, 58 were seropositive; thus the total seroprevalence of hantavirus antibodies in small mammals was 3.14%. The seroprevalence for *O. longicaudatus* was 2.22%, 0.65% for *A. hirta* and 0.27% for *A. olivacea*. The distribution of seropositives is shown in Figure 2. The total relative seropositivity (including all seropositives and all captured species per locality) was higher in Los Queñes, Romeral (1.64) and lower in Paso El León (Cochamó, Los Lagos region) and Colico (Cunco, Araucanía region; 0.06). By administrative region, relative seropositivities were 0.11 (Ñuble region), 0.25 (Valparaíso region), 1.4 (O’Higgins region), 1.28 (Biobío region), 1.42 (Los Lagos region), 1.46 (Araucanía region), 1.39 (Los Ríos region) and 3.03 (El Maule region). Despite HCPS cases reported in Coquimbo and Aysén regions, no seropositive rodents were detected (Table 1). No seropositive rodents were also found in the Metropolitan region despite sampling in 14 localities (Table 1) and registering 54 HCPS cases.

### 3.3. Intraspecific Seropositivity

A total of 58 specimens were found positive for antibodies against the ANDV (seropositives) from 30 localities (Figure 1). Of the 631 *O. longicaudatus* captured, 41 were seropositive from 22 localities (Figure 2A), with an intraspecific seroprevalence of 6.5%. For *A. hirta*, 234 individuals were captured, 12 were seropositive from nine localities (Figure 2B) with an intraspecific seroprevalence of 5.13%. For *A. olivacea*, 636 individuals were captured, with five seropositives from four localities (Figure 2C) and an intraspecific seroprevalence of 0.79%. All seropositive *O. longicaudatus* were adults, including 32 males and nine females. For *A. hirta*, of the 12 seropositives, seven were adult males, four adult females and one juvenile male. For *A. olivacea*, five were seropositive, including three adult males, one juvenile male and one juvenile female.

Among sites, the highest relative seropositivity for *O. longicaudatus* was reported in Las Peñas 2002, San Fernando, O’Higgins region and Los Queñes, Romeral, Maule region (3.6), and the lowest in Paso El León, Cochamó, Los Lagos region and Colico, Cunco, Araucanía region (0.08). *A. hirta* showed the highest relative seropositivity in Caleta Rollizo, Cochamó, Los Lagos region (4.02) and the lowest in Playa San Julián, Corral, Los Ríos region (0.14). *A. olivacea* showed the highest relative seropositivity in Los Queñes, Romeral, Maule region (3.6) and Ucúquer, Litueche, O’Higgins region (0.32).

Of the 631 *O. longicaudatus* captured, 127 had wounds, of which 26 (20.5%) were seropositive to ANDV, including 23 adult males and three adult females. Of the 101 seronegatives with wounds, 90 were adults and 11 juveniles; 55 were males and 46 females. Of the 234 *A. hirta* captured, 67 specimens showed wounds, including five (7.5%) seropositive adults (three males and two females). For the 62 seronegative *A. hirta* with wounds, 51 were adults and 11 were juveniles, and 45 were males and 17 females. Of the 636 *A. olivacea* captured, 91 specimens had wounds, including one seropositive juvenile female and one seropositive adult male. Among the 89 seronegative *A. olivacea* with wounds, there were 82 adults and seven juveniles, and 59 males and 30 females.

### 3.4. Association Between Seropositives and Biological Traits

By using contingency tables, we analyzed the relationships between species (*O. longicaudatus, A. hirta* and *A. olivacea*) versus sex, wounds and age (Table 2) to determine the differences among seropositives. For *O. longicaudatus*, seropositivity was significantly higher in males than in females (*p* = 0.0028), in wounded (*p* < 0.0001) specimens and in adults (*p* = 0.0066). For *A. hirta* and *A. olivacea*, neither sex, wounds nor age were associated with seropositivity.

**Figure 3:**
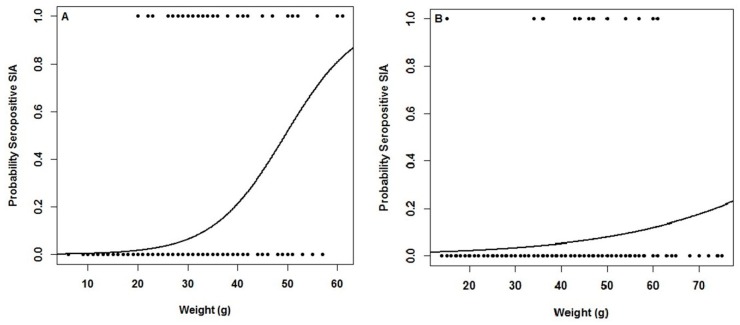
Logistic regression including seropositives for anti-ANDV antibodies versus weight of each capture specimen in sites associated with human hantavirus cases in Chile for (A) *O. longicaudatus*, and (B) *A. hirta*.

Logistic regression (Figure 3A) using serostatus and weight showed that for *O. longicaudatus*, there is an increase in the probability to be seropositive for individuals of higher weight. Individuals of at least 60 g in weight have 80% or higher probability to be seropositive. This probability value drastically decreases with individuals of lower weight (i.e., an individual of 40 g has a 21% probability to be seropositive). Contrasting data were found for *A. hirta* (Figure 3B). In spite of the trend in which the higher the weight higher chances to be seropositive, our study showed that an individual of 70 g has a probability lower than 20% to be seropositive.

### 3.5. Human HCPS Cases

**Figure 4:**
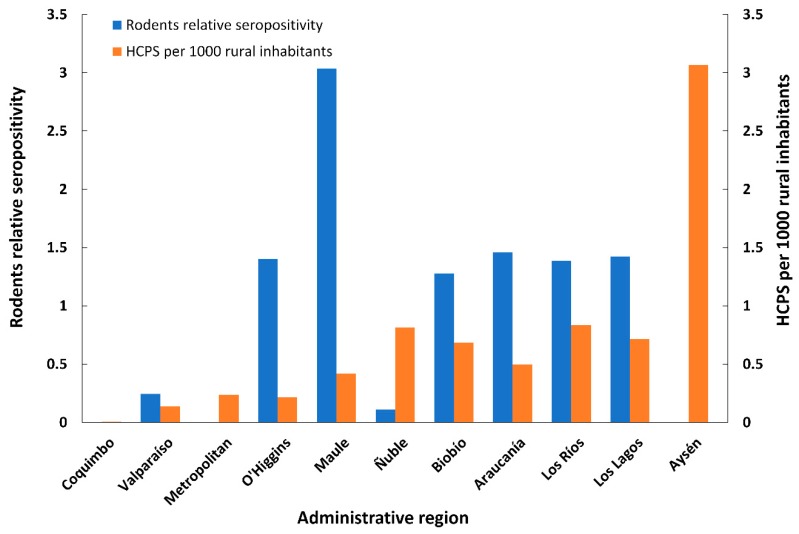
Rodents’ relative seropositivity in sites associated with human Hantavirus cases, and human hantavirus cases per 1000 rural inhabitants across the 11 administrative sampled regions in Chile. Regions are ordered from north (left) to south (right).

During the 19-year study period, 1060 human cases were confirmed as positive for HCPS in the 11 administrative regions in the southcentral regions of Chile sampled in this study (information available from the web page of Ministerio de Salud de Chile, http//:epi.minsal.cl). Rodent relative seropositivity was highest in the Maule region, whereas rates approaching 1.5% were found in O’Higgins, Araucanía, Biobío, Los Ríos and Los Lagos regions. We did not detect seropositive rodents in Coquimbo and Aysén regions (Figure 4), probably due to a bias in the low sampling effort (one sampling in each region). No seropositive rodents were also found in peridomestic sites in the Metropolitan region despite sampling in 14 localities (Table 1) and registering 54 HCPS cases. During the sampling period (2000–2018), the higher number of HCPS cases was registered in Los Lagos region (184 cases), and the lowest in Coquimbo (one case). However, when standardized by people inhabiting in rural areas, human hantavirus cases were notably higher in Aysén region, followed by Los Ríos, Ñuble and Los Lagos (Figure 4).

## 4. Discussion

The extension of the geographical distribution of a zoonotic disease is intrinsically linked to the niche of its host. How hosts interact with human activity and settlements, and the degree of pathogen transmission among host and human populations, will determine the scale of infections that will produce the disease in humans. Hantaviruses are known to be transmitted horizontally [21,40], therefore ecological interactions are key to understanding the spread of the virus among reservoir populations. It is also known that a virus may be harbored by two or more species, with higher opportunities for host switch particularly for sympatric species [41,42,43]. Our study shows the importance of conducting long-term studies that cover a large part of the distribution of a pathogen and its hosts, allowing us to infer patterns of infection and the scale of a host’s epidemiologic importance.

The eleven rodent species analyzed during the nineteen years of sampling in areas associated with human hantavirus cases showed different patterns of abundance in Chile. The highest relative abundance within each sampling site for *O. longicaudatus* was found in the Los Ríos region, for *A. olivacea* and *A. hirta* in the Biobío region, for *A. longipilis* and *Mus musculus* in the Valparaíso region, for *A. sanborni* in the Los Lagos region, for *L. micropus* and *R. rattus* in the Araucanía region and for *P. darwini* and *R. norvegicus* in the O’Higgins region (Table 1, Appendix A). The geographic heterogeneity among rodent abundances is expected and linked to their ecology. For example, despite the wide distribution of *O. longicaudatus* in Chile (from the southern of Atacama desert to Patagonia), the primary habitat of this species occurs in mesic areas that are more prominent between 35–50°S in the Valdivian and Patagonian rain forest ecoregions [36,44]. Our study supports this inference, as more *O. longicaudatus* were captured in localities of Biobío, Araucanía, Los Ríos and Los Lagos regions that fall within the Valdivian rain forests (Appendix A). To understand the scale of the relation between abundance of host and prevalence of the pathogen, we compared the host relative abundance and pathogen infection measured as relative seropositivity. Comparing sampling localities, we found heterogeneous results (Table 1). For example, high abundance of *O. longicaudatus* and absence of seropositives were registered in Vilumanque (Concepción), and Ñancul (Panguipulli), contrasting with localities with low abundance of this species and high seropositivity (e.g., Las Peñas, San Fernando, O’Higgins and La Palma, Valparaíso). This expected pattern was also reported by several studies [20,30], emphasizing the uncertainty of sampling the host carrying the virus despite the epidemiological and ecological analyses to determine the most probable site of human infection [45]. We acknowledge that our estimations of seropositivity might be biased due to an error associated to false positives and/or negatives determined by the SIA method [46]. However, we expect a low error in this parameter [46,47].

In the present study, the seroprevalence of 3.14% for all small mammals and 3.63% for sigmodontine rodents was higher to that reported previously in Chile [20,30,48], an expected result due to our sampling scheme linked to recent confirmed HCPS cases. The variation of total seroprevalence reported in the Americas includes similar percentages to those of our study, such as in Argentina (1.5% in the north, 2.6% in the center and 5.4% in the south) [49], Brazil (2.1%) [50] and North America (average 5%) [51], but differs from others with higher rates such as Panama, Mexico and United States of America [52,53,54,55].

For *O. longicaudatus*, we found an intraspecific seroprevalence of 6.5%, a value within the range of other studies in Chile reporting seroprevalences of 10.4% [20] and 2.5% [30]. The heterogeneity observed in the seroprevalence of rodent populations depends on several factors impacting the ecological dynamics of the host and the virus, such as climate [56], landscape structure [52,57], density-based and/or frequency-based dependence [58,59,60], dilution effect [61], viral recrudescence [47] and habitat disturbance [26,62]. Our sampling scheme also allowed us to show new evidence for the prevalence of antibody against ANDV in Chile in species other than the major host. We found an intraspecific seroprevalence of 5.13% for *A. hirta* and 0.79% for *A. olivacea.* For many years, *A. longipilis* was identified as the second species with higher seroprevalence in Chile. However, new taxonomic rearrangements show that those results were skewed, because the results should be reassigned to *A. hirta* [33,42]. Taking this into account, the seroprevalence for *A. hirta* ranges from 9.3% in Los Ríos region [63] to 1.84% in Los Lagos region [21], representing the highest seroprevalence for hantavirus in Chile after the main reservoir *O. longicaudatus*. Compared with *O. longicaudatus* (6.5%), our seroprevalence data for *A. hirta* (5.13%) acquires new relevance, particularly in understanding its role in maintaining the virus in nature, or potentially as host of a new lineage of hantavirus of unknown pathogenicity [5,36,64]. Based on ours and all previous published data, no *A. longipilis* have been found seropositive to ANDV in Chile. For *A. olivacea*, the most abundant species captured, an intraspecific seroprevalence of 7.5% and 0.73% has been reported in Aysén region [29,65,66], 2.2% in Los Lagos region [66] and 1.5% in Chubut, Argentina [67]. Hantaviruses are capable of infecting multiple host species [5,45], and host-switching events have been reported between species of the same and different families and also separate orders [68]. Cross-species transmission is more likely to occur among sympatric and evolutionary related species, so the study of their ecological interactions is key to understand the processes leading to species jumps and emergence of outbreaks. ANDV experimental transmission was demonstrated from *O. longicaudatus* to *A. olivacea* [21], but the epidemiological importance of *A. hirta* and *A. olivacea* remains unclear [42]. We highlight that our study is one of the few long-term involving only areas associated with human hantavirus cases, therefore the seroprevalences may also differ from others covering alternative sampling designs.

The relationships between species (*O. longicaudatus, A. hirta* and *A. olivacea*) versus sex, wounds and age have been frequently studied to infer major determinants of spread and maintenance of viruses in nature. It has been proposed that most of seropositive *O. longicaudatus* are adult males, which was evaluated in this study. A positive relationship between weight and seropositivity in *O. longicaudatus* suggests that adults are more likely to be infected than juveniles. Although we did not search for viral RNA in seronegative rodents, there it might be a proportion of rodents that are viremic and without antibodies that might be excreting the virus into the environment. We found that adult males (32/41 of positives) are more likely to be seropositive than females (9/41 of positives; Table 2), a result congruent with studies in several wild reservoirs [20,67,69,70,71,72,73]. Our logistic regression (Figure 2) also shows that *O. longicaudatus* weighing 60 g or more were more likely (80%) to be infected with ANDV. A slightly different analysis in the same species in Argentina showed that individuals not injured of 50 g (approx.) and those of 43 g (approx.) with wounds, have a probability of 0.80 to be infected with the virus [73]. Our analysis was performed including both wounded and non-wounded *O. longicaudatus* from all ages. We chose this approach because the role of wounds in hantavirus transmission is still in debate (see below). Adults seem to be more likely to contract the virus because transmission of the virus requires a prolonged period of exposure [74]. *O. longicaudatus* adult females were less likely to be seropositive, suggesting that adult males may have a major role in maintaining the virus transmission in nature. However, the frequency of seropositive females found across several studies is evidence that they may play a more important role than the one currently recognized. Space use and social mating system may contribute to reveal the factors maintaining the virus in wild populations and explaining sex differences of infected hosts [75]. However, viral transmission through aggressive encounters including frequent bites among adult males have been proposed as major hypothesis to explain sex differences [56,76,77,78].

We found 127 *O. longicaudatus* with wounds of which 26 were seropositive (23 males and three females). Our results provide evidence that *O. longicaudatus* adult males with wounds would be more likely to be seropositive (Table 2). An association between wounds and seropositive status suggests that antagonistic encounters between reproductive individuals would be a mechanism of transmission between host populations [79,80,81]. Interestingly, the probability that an *O. longicaudatus* adult male in Argentina is seropositive is five times higher in injured individuals [73]. The previous study showed that adult males with wounds with a body-mass greater than 44g have a probability of 0.84 to be seropositive, while individuals with wounds and a body-mass less than 30 g have a probability of being seropositive of 0.2. Although wounds seem to be the mechanism for intraspecific transmission of hantaviruses, a few studies have found different results. For example, in *Peromyscus maniculatus*, the prevalence seemed to reach a peak much earlier in individuals with wounds, possibly indicating that aggression is not the only mechanism of transmission of *Sin Nombre orthohantavirus* among deer mice [69]. The relationship between hosts with wounds and seropositivity might also be a consequence of the infection [78,80,82,83,84], or it might be related to a greater population density of the reservoir, resulting in viral transmission caused by the greater environmental contamination rather than by aggression among rodents [80]. No relationship between infection and individuals with wounds was found among the *O. longicaudatus* in southern Argentina [67]. There are also social behaviors such as identification, mounting, smelling and persecution that favor physical unaggressive contact among individuals [85]. Field experiments in *O. longicaudatus* do not support a relationship between the presence of wounds and the transmission of ANDV, indicating that other factors such as grooming or aerosol transmission may be playing a major role for efficient viral transmission [21].

Unlike *O. longicaudatus*, seropositive juveniles were identified in the two species of *Abrothrix.* For *A. hirta*, one juvenile male out of 12 was infected. Three adult males and two juveniles (one male and one female) were seropositive for *A. olivacea*. The number of *Abrothrix* infected with hantavirus is frequently low or absent compared to that of sympatric *O. longicaudatus,* both in areas with or without human cases [20,30,86]. In addition to global seroprevalence and sex, we tested if wounds were related to infected *Abrothrix*. We found no association in both species of *Abrothrix* (5/12 seropositives of *A. hirta* and 2/5 for *A. olivacea* with wounds; Table 2), similar to a previous study [63]. Despite some efforts to study viral ecology of coexisting species with the main reservoir, the epidemiological relevance of *Abrothrix* is still unknown [21,29,30,42,87].

The ecological properties of host and vectors are influenced by environmental features [88], which may be strong determinants in the transmission of the virus to humans across different spatial and temporal scales [36,89]. The wide latitudinal range of our samplings ranges from a Mediterranean heterogeneous vegetation mosaic to a mixed evergreen-deciduous temperate and Patagonian forest (30°30’S–45°30’S) [90,91]. These areas are characterized by contrasting landscapes resulting in strong differences in population structure of *O. longicaudatus* [92,93], *A. hirta* [94] and *A. olivacea* [95]. Variability in the population dynamic of *O. longicaudatus* populations across their latitudinal range in Chile may impact transmission and infection rates of ANDV in rodent hosts [36]. Therefore, humans may be differentially exposed to viral infection. A variety of studies reported high differences in seroprevalence of *O. longicaudatus* in Chile across administrative regions, ranging from 23% for the O’Higgins region [66], 12.74% and 12,7% for the Aysén region [29,65], 13.51% in the Los Ríos and Los Lagos regions [63] to 1.44% in the Magallanes region [96]. Interestingly, we found that rodents’ relative seropositivity was higher in the Maule region (around 34–36°S). This region is within the Mediterranean ecoregion, which showed the highest relative seropositivity in Chile [36]. Complex interacting determinants may be involved in explaining this result [97,98]. Landscape structure and hosts’ responses to disturbances and habitat modifications may strongly influence reservoir populations [99] and infectious disease dynamics [98]. For example, hosts may become the dominant species in degraded ecosystems [100], and agroecosystems may play a relevant but still unknown role in harboring infected hosts, and therefore in the transmission of virus to humans [101]. Agriculture and habitat modification, features that characterize the Maule region, are also found in several other regions where we detected high seroprevalence (Figure 4). These inferences acquire more relevance when small mammal studies are linked to areas where humans are at higher risk of contracting the pathogen such as domestic/peridomestic settings [26,49,102]. Similar values of relative seropositivity were found across O’Higgins, Araucanía, Biobío, Los Ríos and Los Lagos regions. We did not detect seropositive rodents in Coquimbo and Aysén regions (Figure 4), probably due to the low comparative sampling effort (one sampling locality in each region). No seropositive rodents were found in peridomestic sites in the Metropolitan region despite sampling in 14 localities (Table 1) and registering 54 confirmed HCPS cases. Whether these cases contracted the infection outside of this region or we were unable to detect seropositives due to our sampling scheme remains to be elucidated. We observed a trend of finding an increased number of HCPS cases from north to south (Figure 4). The high number of reported HCPS cases found the Aysén region was likely influenced by the approach we used (HCPS cases/1000 rural inhabitants). In fact, rural inhabitants in Aysén were significantly lower compared to the other regions reported in this study; also 61 HCPS cases were confirmed in the Aysén region during the study period while a higher number of cases were reported for Los Lagos (*N* = 184), Araucanía (*N* = 158) and Biobío (*N* = 156) regions. Despite the differences in the sampling efforts across administrative regions, the geographic and temporal span of our study highlights the heterogeneity of HCPS cases and the spatial fluctuations of seropositive hosts. Consequently, these data emphasize that integrating ecological understanding of host and pathogens, spatial and temporal surveillance, epidemiology and public health agencies is fundamental to i) understanding the driving processes that ultimately lead to the transmission of virus to humans and ii) successfully designing policies and campaigns to prevent the infection from zoonotic diseases [26,52,103,104].

## 5. Conclusions

Our study assessed how ecology and geography influence host and viral dynamics in areas associated with HCPS cases in Chile. We confirmed the major role of *O. longicaudatus* as a reservoir of ANDV in Chile and the significance of adult males in maintenance and transmission of the virus in nature. Our results indirectly show that wounds might play a significant role in the intraspecific transmission of the virus, which coupled with alternative routes previously reported, suggest that to fully understand the host-viral transmission, further investigation is required on social and behavior interactions of rodents. We highlighted that the epidemiological importance of *A. hirta* and *A. olivacea* in maintaining and/or transmitting the virus among wild populations and to humans remains unclear.

The high variation in rodents’ abundance and seroprevalence across a wide geography, emphasizes the need for i) understanding the drivers that determine the dynamic of fluctuating populations of reservoirs, ii) determining the role of other interacting species for the viral spread and iii) integrating efforts from different disciplines to more accurately determine transmission rates of pathogens within host populations, host switch and ultimately to humans. Elucidating these patterns is critical to prevent future zoonotic outbreaks by implementing effective intervention strategies.

## Figures and Tables

**Table 1:** Relative abundance and relative seropositivity to ANDV by locality and species.

		Relative Abundance											Relative Seropositivity			
Locality, County	Administrative Region	Al	Ah	Ao	As	Am	Lm	Ol	Pd	Mm	Rn	Rr	Ol	Ah	Ao	Total
Chiñigue, Ovalle	Coquimbo	1.00	0.00	2.33	0.00	0.00	0.00	0.00	0.00	0.67	0.67	0.33	0.00	0.00	0.00	0.00
Fundo Chuico Blanco, Hijuelas	Valparaíso	0.00	0.00	0.00	0.00	0.00	0.00	0.37	0.00	0.00	1.11	0.00	0.00	0.00	0.00	0.00
Llaillay, Llaillay	Valparaíso	0.00	0.00	0.42	0.00	0.00	0.00	0.00	0.00	2.53	0.42	0.00	0.00	0.00	0.00	0.00
Lo Mardones, Quillota	Valparaíso	0.00	0.00	2.06	0.00	0.00	0.00	0.48	0.00	0.63	0.95	0.00	0.00	0.00	0.00	0.00
La Palma, Quillota	Valparaíso	0.00	0.00	0.33	0.00	0.00	0.00	0.33	0.00	0.00	0.83	0.17	3.00	0.00	0.00	0.60
Población Prat, Villa Alemana	Valparaíso	0.00	0.00	4.67	0.00	0.00	0.00	0.00	0.00	1.00	0.00	0.33	0.00	0.00	0.00	0.00
Laguna Verde, Valparaíso	Valparaíso	0.83	0.00	0.56	0.00	0.00	0.00	0.00	0.00	0.00	0.56	0.00	0.00	0.00	0.00	0.00
Las Docas, Valparaíso	Valparaíso	3.23	0.00	0.40	0.00	0.00	0.00	1.41	1.01	0.00	0.00	0.00	0.00	0.00	0.00	0.00
Alto el Manzano, TilTil	Metropolitan	0.00	0.00	0.00	0.00	0.00	0.00	0.00	0.00	2.33	0.00	0.00	0.00	0.00	0.00	0.00
San Antonio de Naltahua, Isla de Maipo	Metropolitan	0.00	0.00	0.67	0.00	0.00	0.00	0.00	0.00	0.00	0.00	0.67	0.00	0.00	0.00	0.00
La Reina, La Reina	Metropolitan	0.00	0.00	0.67	0.00	0.00	0.00	0.00	0.00	0.00	0.00	2.00	0.00	0.00	0.00	0.00
Lo Espejo, Lo Espejo	Metropolitan	0.00	0.00	0.50	0.00	0.00	0.00	0.00	0.00	0.33	0.17	0.00	0.00	0.00	0.00	0.00
El Canelo, San José de Maipo	Metropolitan	0.00	0.00	0.00	0.00	0.00	0.00	0.39	0.39	0.00	0.39	0.78	0.00	0.00	0.00	0.00
La Florida Alto, La Florida	Metropolitan	0.37	0.00	3.33	0.00	0.00	0.00	0.00	0.00	0.00	0.00	0.00	0.00	0.00	0.00	0.00
Mallarauco, Melipilla	Metropolitan	0.00	0.00	1.67	0.00	0.00	0.00	0.33	0.00	2.00	0.00	0.00	0.00	0.00	0.00	0.00
San Antonio, San Antonio	Valparaíso	0.00	0.00	0.00	0.00	0.00	0.00	0.44	0.00	1.33	0.00	0.89	0.00	0.00	0.00	0.00
Pomaire, Melipilla	Metropolitan	0.00	0.00	0.89	0.00	0.00	0.00	0.00	0.00	0.00	0.22	0.22	0.00	0.00	0.00	0.00
Constructora Inca, Melipilla	Metropolitan	0.00	0.00	2.56	0.00	0.00	0.00	0.00	0.00	0.32	0.00	0.00	0.00	0.00	0.00	0.00
Condominio Puerta del Sol, Talagante	Metropolitan	0.00	0.00	1.33	0.00	0.00	0.00	0.33	0.00	0.00	0.00	0.00	0.00	0.00	0.00	0.00
Talagante, Talagante	Metropolitan	0.00	0.00	6.67	0.00	0.00	0.00	0.00	0.00	0.42	2.08	0.00	0.00	0.00	0.00	0.00
Chocalán, Melipilla	Metropolitan	0.00	0.00	3.67	0.00	0.00	0.00	0.33	0.00	0.00	0.67	0.00	0.00	0.00	0.00	0.00
Fundo La Ventolera, Santo Domingo	Valparaíso	4.00	0.00	6.67	0.00	0.00	0.00	0.00	0.00	0.00	0.67	0.00	0.00	0.00	0.00	0.00
El Ingenio, San José de Maipo	Metropolitan	0.00	0.00	0.33	0.00	0.00	0.00	0.00	0.00	0.00	0.33	0.00	0.00	0.00	0.00	0.00
Hijuelas, Hijuelas	Valparaíso	0.00	0.00	0.95	0.00	0.00	0.00	0.00	0.00	0.95	0.48	0.00	0.00	0.00	0.00	0.00
Abrantes, Paine	Metropolitan	0.00	0.00	1.00	0.00	0.00	0.00	0.00	0.00	0.33	0.33	1.00	0.00	0.00	0.00	0.00
Ucúquer, Litueche	O’Higgins	0.00	0.00	3.11	0.00	0.00	0.00	1.33	1.33	0.00	0.00	1.11	0.00	0.00	0.32	0.15
Quilamuta-Las Cabras, Las Cabras	O’Higgins	0.33	0.00	1.00	0.00	0.00	0.00	2.67	0.00	0.00	0.67	0.00	0.00	0.00	0.00	0.00
Coya, Machalí	O’Higgins	0.00	0.00	0.69	0.00	0.00	0.00	0.69	1.38	0.00	3.45	0.00	1.45	0.00	0.00	0.16
Sn Vicente de Tagua Tagua, Sn Vicente de Tagua Tagua	O’Higgins	0.00	0.00	0.28	0.00	0.00	0.00	0.00	0.00	0.42	0.00	0.14	0.00	0.00	0.00	0.00
Los Maitenes, Lolol	O’Higgins	0.00	0.00	0.46	0.00	0.00	0.00	0.31	0.00	0.15	0.31	0.00	0.00	0.00	0.00	0.00
Las Peñas, San Fernando 2002	O’Higgins	0.00	0.00	0.56	0.00	0.00	0.00	0.28	0.00	0.00	0.00	0.00	3.60	0.00	0.00	1.20
Las Peñas, San Fernando 2018	O’Higgins	0.00	0.00	1.33	0.00	0.00	0.00	0.00	0.33	0.00	0.50	1.00	0.00	0.00	0.00	0.00
El Sauce, Chimbarongo	O’Higgins	0.00	0.00	2.31	0.00	0.00	0.00	0.77	0.00	0.00	0.00	0.26	1.30	0.00	0.00	0.30
Lipimávida, Vichuquén	Maule	0.44	0.00	0.22	0.00	0.00	0.00	1.78	0.00	0.00	0.00	0.00	0.00	0.00	0.00	0.00
Duao, Licantén	Maule	1.00	0.00	8.00	0.00	0.00	0.00	12.33	0.00	0.00	0.67	0.00	0.32	0.00	0.00	0.18
Escuela Quilpoco, Rauco	Maule	0.00	0.00	0.00	0.00	0.00	0.00	0.37	0.00	0.37	1.11	0.00	2.70	0.00	0.00	0.54
El Pumaitén, Romeral	Maule	0.67	0.00	3.00	0.00	0.00	0.00	0.33	0.00	0.00	0.00	0.67	0.00	0.00	0.00	0.00
Hualañé, Hualañé 2010	Maule	0.00	0.00	0.22	0.00	0.00	0.00	1.33	0.00	0.00	0.00	0.00	0.00	0.00	0.00	0.00
El Trapiche, Curicó	Maule	0.00	0.00	3.00	0.00	0.00	0.00	1.00	0.00	0.33	0.17	0.50	1.00	0.00	0.00	0.20
Hualañé, Hualañé 2012	Maule	0.00	0.00	3.06	0.00	0.00	0.00	0.56	0.00	0.00	0.00	0.00	0.00	0.00	0.00	0.00
Palquibudi, Rauco	Maule	0.00	0.00	0.74	0.00	0.00	0.00	0.37	0.00	0.00	0.37	0.00	0.00	0.00	0.00	0.00
Los Queñes, Romeral	Maule	0.00	0.00	0.28	0.00	0.00	0.00	1.11	0.00	0.00	0.28	1.39	3.60	0.00	3.60	1.64
Las Lomas, San Clemente	Maule	0.00	0.00	0.67	0.00	0.00	0.00	0.00	0.00	0.00	0.00	0.67	0.00	0.00	0.00	0.00
Canal Melado, Longaví	Maule	0.00	0.27	1.87	0.00	0.00	0.00	0.80	0.00	0.00	0.00	0.00	1.25	0.00	0.00	0.34
Retupel, Cauquenes	Maule	0.00	0.00	2.33	0.00	0.00	0.00	1.67	0.00	0.00	0.00	0.00	0.60	0.00	0.00	0.25
Bullileo, Parral	Maule	0.00	0.42	0.00	0.00	0.00	0.00	1.25	0.00	0.00	0.42	1.25	0.00	0.00	0.00	0.00
San Miguel de Ablemo, San Carlos	Ñuble	0.00	0.00	7.10	0.00	0.00	0.00	3.09	0.00	0.00	0.93	1.54	0.00	0.00	0.00	0.00
Tres Esquinas, Coihueco	Biobío	0.00	0.00	0.15	0.00	0.00	0.00	0.00	0.00	0.00	0.00	0.00	0.00	0.00	0.00	0.00
Agua Tendida, Tomé	Biobío	0.00	3.67	5.67	0.00	0.00	0.00	3.33	0.67	0.00	0.33	0.33	0.60	0.27	0.00	0.21
Chillán-Pinto, Chillán	Ñuble	0.00	1.33	7.67	0.00	0.00	0.00	3.00	0.00	0.33	0.33	0.67	0.33	0.00	0.00	0.08
Lloicura, Tomé	Biobío	0.00	1.63	0.00	0.00	0.00	0.00	0.00	0.00	0.00	0.00	0.33	0.00	0.00	0.00	0.00
Peñablanca, Quillón	Ñuble	0.00	0.35	1.39	0.00	0.00	0.00	2.08	0.00	0.00	1.04	0.69	0.00	0.00	0.00	0.00
Vilumanque, Concepción	Biobío	0.00	8.72	8.26	0.00	0.00	0.00	11.93	0.00	0.00	0.00	0.46	0.00	0.00	0.00	0.00
Chaimávida, Concepción	Biobío	0.00	0.44	1.11	0.00	0.00	0.00	2.89	0.00	0.00	0.00	0.00	0.00	0.00	0.00	0.00
Forestal Millalemu, El Carmen	Ñuble	0.00	0.67	0.89	0.00	0.00	0.00	4.22	0.00	0.00	0.00	0.22	0.00	0.00	0.00	0.00
Talcamávida, Hualqui	Biobío	0.00	1.56	0.67	0.00	0.00	0.00	1.56	0.00	0.44	0.00	0.22	0.00	0.00	0.00	0.00
Forestal Millalemu, Tucapel	Biobío	0.00	1.33	7.00	0.00	0.00	0.00	3.00	0.00	0.00	0.00	0.33	0.67	0.00	0.00	0.17
Santa Juana, Santa Juana	Biobío	0.00	0.67	0.00	0.00	0.00	0.00	2.22	0.00	0.00	0.44	0.00	0.00	0.00	0.00	0.00
Los Ángeles, Los Ángeles	Biobío	0.00	0.00	1.28	0.00	0.00	0.00	0.00	0.00	0.00	0.64	0.00	0.00	0.00	0.00	0.00
Cañicura, Quilleco	Biobío	0.00	0.17	3.33	0.00	0.00	0.00	2.83	0.00	0.00	0.50	0.50	1.06	0.00	0.00	0.41
Hacienda San Lorenzo, Quilleco	Biobío	0.00	2.44	2.67	0.00	0.00	0.67	1.33	0.00	0.00	0.00	0.00	0.00	0.00	0.00	0.00
Alto las Viñas, Los Ángeles	Biobío	0.00	0.00	8.67	0.00	0.00	0.00	2.67	0.00	0.33	0.00	0.67	0.00	0.00	0.00	0.00
Antihuala, Los Álamos	Biobío	0.00	1.00	0.33	0.00	0.00	0.00	1.67	0.00	0.00	0.33	2.67	0.00	0.00	0.00	0.00
La Curva, Cañete	Araucanía	0.00	0.17	1.00	0.00	0.00	0.00	0.33	0.00	0.00	1.33	0.00	0.00	0.00	0.00	0.00
Llacolén, Contulmo	Biobío	0.00	0.59	1.04	0.00	0.00	0.00	3.26	0.00	0.00	0.00	0.00	0.00	0.00	0.00	0.00
Llanquén, Lonquimay	Araucanía	0.00	2.00	0.00	0.00	0.00	1.67	1.33	0.00	0.00	0.00	0.00	2.25	0.00	0.00	0.60
Tirúa, Tirúa	Biobío	0.00	0.00	1.11	0.00	0.00	0.00	1.11	0.00	0.00	0.00	0.00	0.00	0.00	0.00	0.00
Lautaro, Lautaro	Araucanía	0.00	0.00	2.00	0.00	0.00	0.00	0.33	0.00	0.67	0.00	0.00	0.00	0.00	0.00	0.00
Fundo La Aguada, Gorbea	Araucanía	0.00	1.56	0.44	0.00	0.00	0.00	0.00	0.00	0.00	0.22	0.22	0.00	1.29	0.00	0.82
Lago Colico, Cunco	Araucanía	0.00	2.19	1.09	0.00	0.00	0.00	12.57	0.00	0.00	0.55	1.09	0.08	0.00	0.00	0.06
Boroa Norte, Toltén	Araucanía	0.00	0.00	0.32	0.00	0.00	0.00	6.13	0.00	0.00	0.32	3.55	0.33	0.00	0.00	0.19
Loncoche, Loncoche	Araucanía	0.00	2.08	1.67	0.00	0.00	0.00	2.50	0.00	0.00	0.00	0.42	0.00	0.00	0.00	0.00
Llancahue Alto, Panguipulli	Los Ríos	0.00	3.83	0.00	0.00	0.00	0.17	0.00	0.00	0.00	0.00	0.00	0.00	0.78	0.00	0.75
Fundo Miraflores, Lanco	Los Ríos	0.00	0.74	1.04	0.00	0.00	0.00	8.74	0.00	0.00	0.00	0.00	0.69	1.35	0.00	0.67
Ñancul, Panguipulli	Los Ríos	0.00	0.00	3.33	0.00	0.00	0.00	17.50	0.00	0.00	0.83	1.67	0.00	0.00	0.00	0.00
Huellelhue-Pishuinco, Valdivia	Los Ríos	0.00	1.63	6.54	0.00	0.00	0.00	0.65	0.00	0.00	0.00	0.33	0.00	0.00	0.00	0.00
Campamento Vientos del Sur, Valdivia	Los Ríos	0.00	1.00	1.00	0.00	0.00	0.00	2.33	0.00	0.00	0.33	1.00	0.43	0.00	0.00	0.18
San Carlos, Corral	Los Ríos	0.00	0.00	3.68	0.00	0.00	0.00	1.47	0.00	0.00	0.74	0.74	0.00	0.00	0.00	0.00
Playa San Julián, Corral	Los Ríos	0.00	7.27	1.52	0.00	0.00	0.00	0.61	0.00	0.00	0.61	0.30	0.00	0.14	0.00	0.10
Fundo Futangue, Lago Ranco	Los Ríos	0.00	3.30	2.20	0.73	0.00	0.00	3.30	0.00	0.00	0.00	0.00	0.00	0.00	0.00	0.00
Las Quemas, Osorno	Los Lagos	0.00	0.88	3.53	0.00	0.29	0.59	0.00	0.00	0.00	0.59	0.00	0.00	1.13	0.57	0.51
Ñilque, Puyehue	Los Lagos	0.00	2.96	1.85	0.00	0.00	0.00	5.56	0.00	0.00	0.37	0.00	0.00	0.34	0.00	0.09
El Encanto, Puyehue	Los Lagos	0.00	2.04	1.67	0.00	1.11	0.00	4.07	0.00	0.00	0.37	0.37	0.00	0.00	0.60	0.10
Rupanquito, Osorno	Los Lagos	0.00	0.97	3.23	0.00	0.00	0.00	8.39	0.00	0.32	0.32	0.65	0.24	0.00	0.00	0.14
El Cabrito, Puerto Octay	Los Lagos	0.00	1.82	2.42	0.00	0.00	0.00	6.06	0.00	0.00	0.30	0.00	0.17	0.55	0.00	0.19
Peulla, Puerto Varas	Los Lagos	0.00	2.22	0.83	0.00	0.00	0.00	0.56	0.00	0.00	0.00	0.83	0.00	0.00	0.00	0.00
Fundo Pichi-Juan, Puerto Varas	Los Lagos	0.00	0.17	1.83	0.00	0.17	0.17	5.33	0.00	0.00	0.00	0.00	0.00	0.00	0.00	0.00
Las Quemas, Puerto Montt	Los Lagos	0.00	0.13	1.43	0.00	0.00	0.13	0.39	0.00	0.00	0.00	0.13	0.00	0.00	0.00	0.00
Caleta Rollizo, Cochamó	Los Lagos	0.00	0.25	3.98	0.00	0.00	0.00	1.99	0.00	0.00	0.00	0.00	0.00	4.02	0.00	0.16
Paso El León, Cochamó	Los Lagos	0.00	4.00	1.00	0.00	0.00	0.00	12.33	0.00	0.00	0.00	0.00	0.08	0.00	0.00	0.06
Parque Nacional Chiloé, Chonchi	Los Lagos	0.00	0.00	4.30	1.04	0.00	0.00	1.04	0.00	0.00	0.15	0.00	0.00	0.00	0.00	0.00
Cerro Negro, Coyhaique	Aysén	0.00	0.56	0.28	0.00	0.00	0.00	0.56	0.00	0.00	0.00	0.00	0.00	0.00	0.00	0.00

Al = Abrothrix longipilis, Ah = Abrothrix hirta, Ao = Abrothrix olivacea, As = Abrothrix sanborni, Am = Abrothrix manni, Lm = Loxodontomys micropus, Ol = Oligoryzomys longicaudatus, Pd = Phyllotis darwini, Mm = Mus musculus, Rn = Rattus norvegicus, Rr = Rattus rattus.

**Table 2:** Results of contingency test of three seropositive species versus sex, age and wounds.

Species/Trait		*O. longicaudatus*						*A. hirta*				*A. olivacea*			
		(+)	(−)	Sum	chi-Square	df	*p*	(+)	(−)	Sum	*p*	(+)	(−)	Sum	*p*
Sex	Male	32	319	351	8.93	1	0.0028	8	129	137	0.394	4	376	380	0.419
	Female	9	271	280				4	93	97		1	255	256	
	Sum	41	590	631				12	222	234		5	631	636	
Age	Adult	41	499	540	7.39	1	0.0066	11	187	198	0.423	3	556	559	0.114
	Juvenile	0	91	91				1	35	36		2	75	77	
	Sum	41	590	631				12	222	234		5	631	636	
Wound	Wounds	26	101	127	51.11	1	<0.0001	5	62	67	0.236	2	89	91	0.152
	Without wounds	15	489	504				7	160	167		3	542	545	
	Sum	41	590	631				12	222	234		5	631	636	

## Appendix A

**Table A1:** Relative abundance, relative seropositivity and seropositives to ANDV by locality and administrative regions of rodent sampled species.

						Relative Abundance											Seropositives			Relative Seropositivity			
Locality, County	Latitude	Longitude	Administrative Region	Total Traps	Year	Al	Ah	Ao	As	Am	Lm	Ol	Pd	Mm	Rn	Rr	Ol	Ah	Ao	Ol	Ah	Ao	Total
Chiñigue, Ovalle	−30.513	−71.102	Coquimbo	300	2006	1.00	0.00	2.33	0.00	0.00	0.00	0.00	0.00	0.67	0.67	0.33	0	0	0	0.00	0.00	0.00	0.00
Fundo Chuico Blanco, Hijuelas	−32.812	−71.089	Valparaíso	270	2002	0.00	0.00	0.00	0.00	0.00	0.00	0.37	0.00	0.00	1.11	0.00	0	0	0	0.00	0.00	0.00	0.00
Llaillay, Llaillay	−32.854	−70.918	Valparaíso	237	2001	0.00	0.00	0.42	0.00	0.00	0.00	0.00	0.00	2.53	0.42	0.00	0	0	0	0.00	0.00	0.00	0.00
Lo Mardones, Quillota	−32.870	−71.221	Valparaíso	630	2016	0.00	0.00	2.06	0.00	0.00	0.00	0.48	0.00	0.63	0.95	0.00	0	0	0	0.00	0.00	0.00	0.00
La Palma, Quillota	−32.908	−71.206	Valparaíso	600	2013	0.00	0.00	0.33	0.00	0.00	0.00	0.33	0.00	0.00	0.83	0.17	1	0	0	3.00	0.00	0.00	0.60
Población Prat, Villa Alemana	−33.070	−71.357	Valparaíso	300	2001	0.00	0.00	4.67	0.00	0.00	0.00	0.00	0.00	1.00	0.00	0.33	0	0	0	0.00	0.00	0.00	0.00
Laguna Verde, Valparaíso	−33.115	−71.666	Valparaíso	360	2006	0.83	0.00	0.56	0.00	0.00	0.00	0.00	0.00	0.00	0.56	0.00	0	0	0	0.00	0.00	0.00	0.00
Las Docas, Valparaíso	−33.138	−71.703	Valparaíso	495	2018	3.23	0.00	0.40	0.00	0.00	0.00	1.41	1.01	0.00	0.00	0.00	0	0	0	0.00	0.00	0.00	0.00
Alto el Manzano, TilTil	−33.165	−70.790	Metropolitan	300	2010	0.00	0.00	0.00	0.00	0.00	0.00	0.00	0.00	2.33	0.00	0.00	0	0	0	0.00	0.00	0.00	0.00
San Antonio de Naltahua, Isla de Maipo	−33.436	−71.021	Metropolitan	300	2004	0.00	0.00	0.67	0.00	0.00	0.00	0.00	0.00	0.00	0.00	0.67	0	0	0	0.00	0.00	0.00	0.00
La Reina, La Reina	−33.456	−70.517	Metropolitan	150	2003	0.00	0.00	0.67	0.00	0.00	0.00	0.00	0.00	0.00	0.00	2.00	0	0	0	0.00	0.00	0.00	0.00
Lo Espejo, Lo Espejo	−33.543	−70.696	Metropolitan	600	2013	0.00	0.00	0.50	0.00	0.00	0.00	0.00	0.00	0.33	0.17	0.00	0	0	0	0.00	0.00	0.00	0.00
El Canelo, San José de Maipo	−33.556	−70.454	Metropolitan	255	2004	0.00	0.00	0.00	0.00	0.00	0.00	0.39	0.39	0.00	0.39	0.78	0	0	0	0.00	0.00	0.00	0.00
La Florida Alto, La Florida	−33.564	−70.532	Metropolitan	270	2005	0.37	0.00	3.33	0.00	0.00	0.00	0.00	0.00	0.00	0.00	0.00	0	0	0	0.00	0.00	0.00	0.00
Mallarauco, Melipilla	−33.569	−71.180	Metropolitan	300	2012	0.00	0.00	1.67	0.00	0.00	0.00	0.33	0.00	2.00	0.00	0.00	0	0	0	0.00	0.00	0.00	0.00
San Antonio, San Antonio	−33.643	−71.563	Valparaíso	225	2001	0.00	0.00	0.00	0.00	0.00	0.00	0.44	0.00	1.33	0.00	0.89	0	0	0	0.00	0.00	0.00	0.00
Pomaire, Melipilla	−33.650	−71.179	Metropolitan	450	2006	0.00	0.00	0.89	0.00	0.00	0.00	0.00	0.00	0.00	0.22	0.22	0	0	0	0.00	0.00	0.00	0.00
Constructora Inca, Melipilla	−33.673	−71.180	Metropolitan	312	2013	0.00	0.00	2.56	0.00	0.00	0.00	0.00	0.00	0.32	0.00	0.00	0	0	0	0.00	0.00	0.00	0.00
Condominio Puerta del Sol, Talagante	−33.677	−70.845	Metropolitan	300	2006	0.00	0.00	1.33	0.00	0.00	0.00	0.33	0.00	0.00	0.00	0.00	0	0	0	0.00	0.00	0.00	0.00
Talagante, Talagante	−33.681	−70.877	Metropolitan	240	2001	0.00	0.00	6.67	0.00	0.00	0.00	0.00	0.00	0.42	2.08	0.00	0	0	0	0.00	0.00	0.00	0.00
Chocalán, Melipilla	−33.735	−71.217	Metropolitan	300	2003	0.00	0.00	3.67	0.00	0.00	0.00	0.33	0.00	0.00	0.67	0.00	0	0	0	0.00	0.00	0.00	0.00
Fundo La Ventolera, Santo Domingo	−33.743	−71.652	Valparaíso	300	2002	4.00	0.00	6.67	0.00	0.00	0.00	0.00	0.00	0.00	0.67	0.00	0	0	0	0.00	0.00	0.00	0.00
El Ingenio, San José de Maipo	−33.786	−70.248	Metropolitan	300	2002	0.00	0.00	0.33	0.00	0.00	0.00	0.00	0.00	0.00	0.33	0.00	0	0	0	0.00	0.00	0.00	0.00
Hijuelas, Hijuelas	−33.813	−71.147	Valparaíso	210	2001	0.00	0.00	0.95	0.00	0.00	0.00	0.00	0.00	0.95	0.48	0.00	0	0	0	0.00	0.00	0.00	0.00
Abrantes, Paine	−33.868	−70.837	Metropolitan	300	2004	0.00	0.00	1.00	0.00	0.00	0.00	0.00	0.00	0.33	0.33	1.00	0	0	0	0.00	0.00	0.00	0.00
Ucúquer, Litueche	−33.998	−71.650	O’Higgins	450	2013	0.00	0.00	3.11	0.00	0.00	0.00	1.33	1.33	0.00	0.00	1.11	0	0	1	0.00	0.00	0.32	0.15
Quilamuta-Las Cabras, Las Cabras	−34.070	−71.300	O’Higgins	300	2002	0.33	0.00	1.00	0.00	0.00	0.00	2.67	0.00	0.00	0.67	0.00	0	0	0	0.00	0.00	0.00	0.00
Coya, Machalí	−34.190	−70.534	O’Higgins	145	2002	0.00	0.00	0.69	0.00	0.00	0.00	0.69	1.38	0.00	3.45	0.00	1	0	0	1.45	0.00	0.00	0.16
San Vicente de Tagua Tagua, San Vicente de Tagua Tagua	−34.433	−71.069	O’Higgins	714	2011	0.00	0.00	0.28	0.00	0.00	0.00	0.00	0.00	0.42	0.00	0.14	0	0	0	0.00	0.00	0.00	0.00
Los Maitenes, Lolol	−34.671	−71.554	O’Higgins	652	2017	0.00	0.00	0.46	0.00	0.00	0.00	0.31	0.00	0.15	0.31	0.00	0	0	0	0.00	0.00	0.00	0.00
Las Peñas, San Fernando 2002	−34.766	−70.776	O’Higgins	360	2002	0.00	0.00	0.56	0.00	0.00	0.00	0.28	0.00	0.00	0.00	0.00	1	0	0	3.60	0.00	0.00	1.20
Las Peñas, San Fernando 2018	−34.767	−70.776	O’Higgins	600	2018	0.00	0.00	1.33	0.00	0.00	0.00	0.00	0.33	0.00	0.50	1.00	0	0	0	0.00	0.00	0.00	0.00
El Sauce, Chimbarongo	−34.815	−70.994	O’Higgins	390	2012	0.00	0.00	2.31	0.00	0.00	0.00	0.77	0.00	0.00	0.00	0.26	1	0	0	1.30	0.00	0.00	0.30
Lipimávida, Vichuquén	−34.870	−72.147	Maule	450	2010	0.44	0.00	0.22	0.00	0.00	0.00	1.78	0.00	0.00	0.00	0.00	0	0	0	0.00	0.00	0.00	0.00
Duao, Licantén	−34.882	−72.154	Maule	300	2003	1.00	0.00	8.00	0.00	0.00	0.00	12.33	0.00	0.00	0.67	0.00	4	0	0	0.32	0.00	0.00	0.18
Escuela Quilpoco, Rauco	−34.955	−71.346	Maule	270	2018	0.00	0.00	0.00	0.00	0.00	0.00	0.37	0.00	0.37	1.11	0.00	1	0	0	2.70	0.00	0.00	0.54
El Pumaitén, Romeral	−34.967	−71.125	Maule	300	2002	0.67	0.00	3.00	0.00	0.00	0.00	0.33	0.00	0.00	0.00	0.67	0	0	0	0.00	0.00	0.00	0.00
Hualañé, Hualañé 2010	−34.971	−71.833	Maule	450	2010	0.00	0.00	0.22	0.00	0.00	0.00	1.33	0.00	0.00	0.00	0.00	0	0	0	0.00	0.00	0.00	0.00
El Trapiche, Curicó	−34.983	−71.317	Maule	600	2004	0.00	0.00	3.00	0.00	0.00	0.00	1.00	0.00	0.33	0.17	0.50	1	0	0	1.00	0.00	0.00	0.20
Hualañé, Hualañé 2012	−34.990	−71.847	Maule	360	2012	0.00	0.00	3.06	0.00	0.00	0.00	0.56	0.00	0.00	0.00	0.00	0	0	0	0.00	0.00	0.00	0.00
Palquibudi, Rauco	−35.030	−71.549	Maule	270	2002	0.00	0.00	0.74	0.00	0.00	0.00	0.37	0.00	0.00	0.37	0.00	0	0	0	0.00	0.00	0.00	0.00
Los Queñes, Romeral	−35.125	−70.773	Maule	360	2012	0.00	0.00	0.28	0.00	0.00	0.00	1.11	0.00	0.00	0.28	1.39	4	0	1	3.60	0.00	3.60	1.64
Las Lomas, San Clemente	−35.484	−71.227	Maule	300	2002	0.00	0.00	0.67	0.00	0.00	0.00	0.00	0.00	0.00	0.00	0.67	0	0	0	0.00	0.00	0.00	0.00
Canal Melado, Longaví	−36.017	−71.501	Maule	375	2002	0.00	0.27	1.87	0.00	0.00	0.00	0.80	0.00	0.00	0.00	0.00	1	0	0	1.25	0.00	0.00	0.34
Retupel, Cauquenes	−36.018	−72.462	Maule	300	2003	0.00	0.00	2.33	0.00	0.00	0.00	1.67	0.00	0.00	0.00	0.00	1	0	0	0.60	0.00	0.00	0.25
Bullileo, Parral	−36.289	−71.413	Maule	240	2001	0.00	0.42	0.00	0.00	0.00	0.00	1.25	0.00	0.00	0.42	1.25	0	0	0	0.00	0.00	0.00	0.00
San Miguel de Ablemo, San Carlos	−36.452	−71.990	Ñuble	324	2003	0.00	0.00	7.10	0.00	0.00	0.00	3.09	0.00	0.00	0.93	1.54	0	0	0	0.00	0.00	0.00	0.00
Tres Esquinas, Coihueco	−36.556	−71.766	Biobío	675	2013	0.00	0.00	0.15	0.00	0.00	0.00	0.00	0.00	0.00	0.00	0.00	0	0	0	0.00	0.00	0.00	0.00
Agua Tendida, Tomé	−36.641	−72.796	Biobío	300	2004	0.00	3.67	5.67	0.00	0.00	0.00	3.33	0.67	0.00	0.33	0.33	2	1	0	0.60	0.27	0.00	0.21
Chillán-Pinto, Chillán	−36.651	−71.999	Ñuble	300	2003	0.00	1.33	7.67	0.00	0.00	0.00	3.00	0.00	0.33	0.33	0.67	1	0	0	0.33	0.00	0.00	0.08
Lloicura, Tomé	−36.689	−72.762	Biobío	306	2003	0.00	1.63	0.00	0.00	0.00	0.00	0.00	0.00	0.00	0.00	0.33	0	0	0	0.00	0.00	0.00	0.00
Peñablanca, Quillón	−36.713	−72.572	Ñuble	288	2001	0.00	0.35	1.39	0.00	0.00	0.00	2.08	0.00	0.00	1.04	0.69	0	0	0	0.00	0.00	0.00	0.00
Vilumanque, Concepción	−36.784	−73.012	Biobío	218	2003	0.00	8.72	8.26	0.00	0.00	0.00	11.93	0.00	0.00	0.00	0.46	0	0	0	0.00	0.00	0.00	0.00
Chaimávida, Concepción	−36.799	−72.952	Biobío	450	2009	0.00	0.44	1.11	0.00	0.00	0.00	2.89	0.00	0.00	0.00	0.00	0	0	0	0.00	0.00	0.00	0.00
Forestal Millalemu, El Carmen	−36.931	−71.744	Ñuble	450	2005	0.00	0.67	0.89	0.00	0.00	0.00	4.22	0.00	0.00	0.00	0.22	0	0	0	0.00	0.00	0.00	0.00
Talcamávida, Hualqui	−37.142	−72.908	Biobío	450	2005	0.00	1.56	0.67	0.00	0.00	0.00	1.56	0.00	0.44	0.00	0.22	0	0	0	0.00	0.00	0.00	0.00
Forestal Millalemu, Tucapel	−37.241	−71.794	Biobío	300	2002	0.00	1.33	7.00	0.00	0.00	0.00	3.00	0.00	0.00	0.00	0.33	2	0	0	0.67	0.00	0.00	0.17
Santa Juana, Santa Juana	−37.342	−72.932	Biobío	450	2009	0.00	0.67	0.00	0.00	0.00	0.00	2.22	0.00	0.00	0.44	0.00	0	0	0	0.00	0.00	0.00	0.00
Los Ángeles, Los Ángeles	−37.463	−72.303	Biobío	312	2000	0.00	0.00	1.28	0.00	0.00	0.00	0.00	0.00	0.00	0.64	0.00	0	0	0	0.00	0.00	0.00	0.00
Cañicura, Quilleco	−37.541	−72.688	Biobío	600	2018	0.00	0.17	3.33	0.00	0.00	0.00	2.83	0.00	0.00	0.50	0.50	3	0	0	1.06	0.00	0.00	0.41
Hacienda San Lorenzo, Quilleco	−37.546	−71.463	Biobío	450	2005	0.00	2.44	2.67	0.00	0.00	0.67	1.33	0.00	0.00	0.00	0.00	0	0	0	0.00	0.00	0.00	0.00
Alto las Viñas, Los Ángeles	−37.559	−72.544	Biobío	300	2004	0.00	0.00	8.67	0.00	0.00	0.00	2.67	0.00	0.33	0.00	0.67	0	0	0	0.00	0.00	0.00	0.00
Antihuala, Los Álamos	−37.706	−73.419	Biobío	300	2006	0.00	1.00	0.33	0.00	0.00	0.00	1.67	0.00	0.00	0.33	2.67	0	0	0	0.00	0.00	0.00	0.00
La Curva, Cañete	−37.796	−73.308	Araucanía	600	2013	0.00	0.17	1.00	0.00	0.00	0.00	0.33	0.00	0.00	1.33	0.00	0	0	0	0.00	0.00	0.00	0.00
Llacolén, Contulmo	−37.916	−73.273	Biobío	675	2013	0.00	0.59	1.04	0.00	0.00	0.00	3.26	0.00	0.00	0.00	0.00	0	0	0	0.00	0.00	0.00	0.00
Llanquén, Lonquimay	−38.150	−71.300	Araucanía	600	2014	0.00	2.00	0.00	0.00	0.00	1.67	1.33	0.00	0.00	0.00	0.00	3	0	0	2.25	0.00	0.00	0.60
Tirúa, Tirúa	−38.272	−73.513	Biobío	270	2006	0.00	0.00	1.11	0.00	0.00	0.00	1.11	0.00	0.00	0.00	0.00	0	0	0	0.00	0.00	0.00	0.00
Lautaro, Lautaro	−38.527	−72.429	Araucanía	300	2002	0.00	0.00	2.00	0.00	0.00	0.00	0.33	0.00	0.67	0.00	0.00	0	0	0	0.00	0.00	0.00	0.00
Fundo La Aguada, Gorbea	−39.060	−72.400	Araucanía	450	2005	0.00	1.56	0.44	0.00	0.00	0.00	0.00	0.00	0.00	0.22	0.22	0	2	0	0.00	1.29	0.00	0.82
Lago Colico, Cunco	−39.064	−71.973	Araucanía	183	2008	0.00	2.19	1.09	0.00	0.00	0.00	12.57	0.00	0.00	0.55	1.09	1	0	0	0.08	0.00	0.00	0.06
Boroa Norte, Toltén	−39.286	−73.076	Araucanía	310	2013	0.00	0.00	0.32	0.00	0.00	0.00	6.13	0.00	0.00	0.32	3.55	2	0	0	0.33	0.00	0.00	0.19
Loncoche, Loncoche	−39.371	−72.612	Araucanía	240	2003	0.00	2.08	1.67	0.00	0.00	0.00	2.50	0.00	0.00	0.00	0.42	0	0	0	0.00	0.00	0.00	0.00
Llancahue Alto, Panguipulli	−39.527	−71.813	Los Ríos	600	2014	0.00	3.83	0.00	0.00	0.00	0.17	0.00	0.00	0.00	0.00	0.00	0	3	0	0.00	0.78	0.00	0.75
Fundo Miraflores, Lanco	−39.543	−72.630	Los Ríos	675	2013	0.00	0.74	1.04	0.00	0.00	0.00	8.74	0.00	0.00	0.00	0.00	6	1	0	0.69	1.35	0.00	0.67
Ñancul, Panguipulli	−39.729	−72.402	Los Ríos	120	2001	0.00	0.00	3.33	0.00	0.00	0.00	17.50	0.00	0.00	0.83	1.67	0	0	0	0.00	0.00	0.00	0.00
Huellelhue-Pishuinco, Valdivia	−39.806	−73.069	Los Ríos	306	2003	0.00	1.63	6.54	0.00	0.00	0.00	0.65	0.00	0.00	0.00	0.33	0	0	0	0.00	0.00	0.00	0.00
Campamento Vientos del Sur, Valdivia	−39.824	−73.204	Los Ríos	300	2003	0.00	1.00	1.00	0.00	0.00	0.00	2.33	0.00	0.00	0.33	1.00	1	0	0	0.43	0.00	0.00	0.18
San Carlos, Corral	−39.862	−73.444	Los Ríos	136	2011	0.00	0.00	3.68	0.00	0.00	0.00	1.47	0.00	0.00	0.74	0.74	0	0	0	0.00	0.00	0.00	0.00
Playa San Julián, Corral	−39.905	−73.379	Los Ríos	330	2011	0.00	7.27	1.52	0.00	0.00	0.00	0.61	0.00	0.00	0.61	0.30	0	1	0	0.00	0.14	0.00	0.10
Fundo Futangue, Lago Ranco	−40.365	−72.315	Los Ríos	273	2002	0.00	3.30	2.20	0.73	0.00	0.00	3.30	0.00	0.00	0.00	0.00	0	0	0	0.00	0.00	0.00	0.00
Las Quemas, Osorno	−40.630	−73.115	Los Lagos	340	2017	0.00	0.88	3.53	0.00	0.29	0.59	0.00	0.00	0.00	0.59	0.00	0	1	2	0.00	1.13	0.57	0.51
Ñilque, Puyehue	−40.725	−72.434	Los Lagos	270	2004	0.00	2.96	1.85	0.00	0.00	0.00	5.56	0.00	0.00	0.37	0.00	0	1	0	0.00	0.34	0.00	0.09
El Encanto, Puyehue	−40.760	−72.444	Los Lagos	540	2017	0.00	2.04	1.67	0.00	1.11	0.00	4.07	0.00	0.00	0.37	0.37	0	0	1	0.00	0.00	0.60	0.10
Rupanquito, Osorno	−40.772	−72.783	Los Lagos	310	2013	0.00	0.97	3.23	0.00	0.00	0.00	8.39	0.00	0.32	0.32	0.65	2	0	0	0.24	0.00	0.00	0.14
El Cabrito, Puerto Octay	−40.909	−72.410	Los Lagos	330	2011	0.00	1.82	2.42	0.00	0.00	0.00	6.06	0.00	0.00	0.30	0.00	1	1	0	0.17	0.55	0.00	0.19
Peulla, Puerto Varas	−41.086	−72.018	Los Lagos	360	2006	0.00	2.22	0.83	0.00	0.00	0.00	0.56	0.00	0.00	0.00	0.83	0	0	0	0.00	0.00	0.00	0.00
Fundo Pichi-Juan, Puerto Varas	−41.243	−72.722	Los Lagos	600	2013	0.00	0.17	1.83	0.00	0.17	0.17	5.33	0.00	0.00	0.00	0.00	0	0	0	0.00	0.00	0.00	0.00
Las Quemas, Puerto Montt	−41.416	−73191.000	Los Lagos	771	2002	0.00	0.13	1.43	0.00	0.00	0.13	0.39	0.00	0.00	0.00	0.13	0	0	0	0.00	0.00	0.00	0.00
Caleta Rollizo, Cochamó	−41.430	−72.308	Los Lagos	402	2011	0.00	0.25	3.98	0.00	0.00	0.00	1.99	0.00	0.00	0.00	0.00	0	1	0	0.00	4.02	0.00	0.16
Paso El León, Cochamó	−41.532	−71.831	Los Lagos	300	2006	0.00	4.00	1.00	0.00	0.00	0.00	12.33	0.00	0.00	0.00	0.00	1	0	0	0.08	0.00	0.00	0.06
Parque Nacional Chiloé, Chonchi	−42.622	−74.106	Los Lagos	675	2013	0.00	0.00	4.30	1.04	0.00	0.00	1.04	0.00	0.00	0.15	0.00	0	0	0	0.00	0.00	0.00	0.00
Cerro Negro, Coyhaique	−45.583	−71.940	Aysén	360	2004	0.00	0.56	0.28	0.00	0.00	0.00	0.56	0.00	0.00	0.00	0.00	0	0	0	0.00	0.00	0.00	0.00

Al = Abrothrix longipilis, Ah = Abrothrix hirta, Ao = Abrothrix olivacea, As = Abrothrix sanborni, Am = Abrothrix manni, Lm = Loxodontomys micropus, Ol = Oligoryzomys longicaudatus, Pd = Phyllotis darwini, Mm = Mus musculus, Rn = Rattus norvegicus, Rr = Rattus rattus
